# Predictors of HPV incidence and clearance in a cohort of Brazilian HIV-infected women

**DOI:** 10.1371/journal.pone.0185423

**Published:** 2017-10-05

**Authors:** Ana Gabriela Travassos, Eduardo Netto, Eveline Xavier-Souza, Isabella Nóbrega, Karina Adami, Maiara Timbó, Karen Abbehusen, Sheyla Fernandes, Camila Duran, Tatiana Haguihara, Fábio Ferreira, Carlos Brites

**Affiliations:** 1 Centro Especializado em Diagnóstico, Assistência e Pesquisa (CEDAP), Secretaria de Saúde do Estado da Bahia, Salvador, Bahia, Brazil; 2 Colegiado de Medicina, Departamento de Ciências da Vida, Universidade Estadual da Bahia, Salvador, Bahia, Brazil; 3 Faculdade de Medicina, Universidade Federal da Bahia, Salvador, Bahia, Brazil; 4 Escola Bahiana de Medicina e Saúde Pública, Salvador, Bahia, Brazil; 5 Laboratório Central de Saúde Pública Prof. Gonçalo Moniz, Salvador, Bahia, Brazil; University of Washington Department of Global Health, UNITED STATES

## Abstract

Persistent infection with high-risk human papillomavirus (HR-HPV) is necessary for the development of precursor lesions and cervical cancer. HPV infection among women living with HIV/AIDS (WLHA) occurs more frequently, presents a higher rate of persistent infections and an earlier progression to cancer. We aimed to evaluate HR-HPV prevalence, incidence and clearance, and its association with HIV viral suppression, immunological response and other risk factors among WLHA followed at an STD/HIV reference center. This was a cohort study conducted at a reference center for STD/AIDS in Northeastern Brazil from September 2013 to September 2015. Follow-up visits were conducted at 6 and 12 months after enrolment, where socio-epidemiological data were obtained. Cervical samples were collected for conventional cytology and HPV DNA research (PCR COBAS® Roche) in addition to blood samples for CD4+ T lymphocyte count and HIV viral load. We prospectively evaluated 333 women. HR-HPV DNA prevalence was 33.3% at baseline. HPV-16 was present in 5.1%, HPV-18 in 3.9% and 29.4% WLHA had other HR-HPV (31, 33, 35, 39, 45, 51, 52, 56, 58, 59, 66 and 68). The HR-HPV incidence during the follow-up was 10.8%, at the 6-month visit was 7.7% and at the 12-month visit was 3.7%. Variables associated with HR-HPV incidence were: nulliparity, combined oral contraceptive use and detectable HIV viral load. The HR-HPV clearance rate was 41.7% and was associated with age >30 years and lymphocyte T CD4 count >500 cells/mm3 at enrolment. These findings contribute to the knowledge about a group of women that need more careful HPV screening and describe the association between an efficient immunological response and HIV viral suppression with lower incidence and increased clearance of HR-HPV.

## Introduction

Cervical cancer was considered an AIDS-defining disease by the CDC in 1993 [1]. HPV infection occurs more frequently in women living with HIV/AIDS (WLHA), and with a higher number of multiple high-risk viral types compared to the general population [2]. Immunological deficiency is another risk factor and, while allowing higher persistence of HPV, it induces the development of lesions at sites other than the cervix, such as the vagina, vulva and anus, which are more frequent and difficult to treat [3]. The progression from a high-grade lesion to cancer is faster in WLHA than in the general population. Although the mechanisms involved in the rate of progression in these women is unclear, the continuous reduction in cellular immunity and the presence of chronic inflammation play a decisive role in this process [4].

Persistence of high-risk HPV (HR-HPV) infection is necessary for the development of cervical cancer and precursor lesions [5]. Genomic integration, chromosomal instability, and interference of the E6 and E7 HPV genes in cell cycle regulation are necessary steps for viral oncogenesis in the genital squamous epithelium [6]. In about 80% of HPV-infected women, viral clearance occurs prior to viral integration into the host genome, the stage responsible for carcinogenesis [7]. The use of combined oral contraceptives, smoking, early sexual debut, multiparity and deficiencies in the immune system are well-established cofactors in the development of cervical cancer [8].

The use of antiretroviral (ART) has improved the quality of life of WLHA; however, disease-free survival and reductions in other HIV-related neoplasias are not reflected in the decrease in the incidence of cervical cancer [9]. Some authors attribute the late onset of ART and the prior genomic integration of HPV to the persistence of HPV infection and evolution to cancer precursor lesion [10]. Adherence to ART and viral suppression seem to be the most important factors in the decrease of HPV infection in WLHA [11,12]. The aim of this study was to evaluate HR-HPV infection, clearance, and the association with HIV viral suppression, the immune response, and other socio-demographic factors in WLHA followed at a referral service in northeastern Brazil.

## Methods

### Patients and settings

This was a prospective cohort study which included women living with HIV/AIDS attending at Centro Especializado em Diagnóstico, Assistência e Pesquisa (CEDAP), carried out from September 2013 to September 2015. CEDAP is a Specialized Assistance Service (SAE) for sexually transmitted infections (STI/HIV) of the city of Salvador, capital of the state of Bahia, Brazil. This service take care to about 60% of the people living with HIV in the state, with approximately 3,500 patients using ART. The WLHA are screened annually with Pap and colposcopy to cervical cancer in this service.

In this study, WLHA undergoing regular follow-up in the gynecology outpatient service of CEDAP were sequentially invited to enroll, even if they did not present signs or symptoms. Recruitment was performed until a sample of 338 women was obtained, which was previously calculated sample size estimating 18% loss to follow-up. WLHA seeing the Professor Edgar Santos University Hospital (HUPES) were also invited to participate in the study and, after inclusion in the project, they were followed up at the gynecological service of CEDAP.

Women who have or had a sex life, previous to the diagnosis of HIV [13] and in follow-up with the gynecological service of CEDAP were included in the study, including those who were pregnant. Exclusion criteria comprised pregnant with obstetric complications, women with previous hysterectomy, genital bleeding at the time of the examination, and antibiotic use in the 30 days preceding the appointment.

### Data collection and laboratory tests

Sociodemographic and clinical data were obtained through interviews with standardized questionnaire (S1 Appendix) during medical appointment. Laboratory tests and interviews were scheduled to be performed at the inclusion of the participants and at the 6-month and 12-month appointments after inclusion. Follow-up visits were considered valid when the participants returned up to 4 months after the expected follow-up visit period.

In each follow-up evaluation, cytologic smears were performed using cervicovaginal samples which were collected with an endocervical brush and an Ayre spatula. The smears were stained according to the recommendations of the Health Ministry of Brazil (HMB) [14] and were analyzed using the Bethesda system [15] by two cytologists of the service, who were blinded to the use of ART. For HPV screening, a cervical sample was collected with a cytobrush, processed through a sample collection kit (Roche) and the presence of HPV-DNA was analyzed using a Cobas® PCR machine (Roche, Mannheim, Germany). The Cobas HPV test detects HPV 16 and HPV 18 individually and, simultaneously, identifies a pool of 12 high‐risk genotypes (31, 33, 35, 39, 45, 51, 52, 56, 58, 59, 66, and 68). In addition to the HPV screening, screening was also performed for *Chlamydia trachomatis* (CT) and *Neisseria gonorrhoeae* (NG) using the Cobas® PCR Media Female for processing; screening was performed by qPCR using a closed in vitro diagnostic (IVD) Cobas 4800® system (Roche, Mannheim, Germany). PCR tests were processed in the Public Health Central Laboratory Professor Gonçalo Moniz (LACEN-Bahia), Brazil.

All participants in the study had a colposcopy performed at each follow-up visit and findings were documented using the International Federation for Cervical Pathology and Colposcopy terminology [16]. Biopsy of the uterine cervix was performed when necessary, as recommended by the HMB [17].

All patients had a blood sample taken at the time of the appointment to evaluate HIV infection through HIV viral load (VL) and CD4+/CD8+ T cell counts. HIV VL was quantified using real-time PCR (Abbot Molecular, Illinois, USA) and CD4+/CD8+ T cell counts were performed by flow cytometry (FACSCalibur, Becton and Dickinson, California, USA); both were performed at the HUPES.

### Endpoints

The prevalence of HR-HPV infection in the study was defined as positive screening for HR-HPV at baseline. Incident infections were subjects with negative inclusion screening and a subsequent positive screening for HR-HPV. The cumulative incidences were not evaluated, were considered only the moment of the first positive result. Clearance of HPV infection was defined as a negative HPV-DNA after being positive on the previous screening. Multiple-type infection was considered when a simultaneous infection with HPV 16 and HPV 18 or one of the HR-HPV pool types was identified.

### Ethical aspects

This study was approved by the Ethics Committee of the Climério de Oliveira Maternity/Federal University of Bahia (process 125,689). Before enrolment, written informed consent was provided by all patients; when they were under 18 years of age, the legal guardian also provided consent. Patients who had any diagnosed infection were rescheduled for treatment according to the HMB’s therapeutic protocol [18]. During study enrolment, the participants were educated about the consequences of HIV, HPV, and other sexually transmitted infections (STIs) and oriented about prevention in each visit.

### Statistical analysis

The sample size of WLHA was estimated using the software Epi Info^TM^ (Version 6.04d.), based on a previous study performed in Brazil[19]. Considering an expected HPV frequency of 50% and absolute precision of 5% in WLHA, a statistical power of 80% and an α level of 0.05, the calculated number of patients for the study was 306 WLHA.

Data analysis was performed using the software SPSS 20.0 (SPSS Inc., Chicago, IL, USA). The statistical analysis of prevalence data was made with Pearson’s chi-squared and Fisher test for the univariate analysis of categorical variables such as marital status (single vs. married/stable union), schooling (<8 years of study vs. ≥8 years of study), ethnicity (white vs. non-white), drug use (yes vs. no), condom use (regular vs. irregular), smoking (yes vs. no), previous condyloma (yes vs. no), use of oral contraceptives (yes vs. no), and ART use (yes vs. no). Continuous variables such as age, number of partners, duration of ART and time since HIV diagnosis were described and transformed to categorical variables as age (≤30 vs > 30 years old), CD4 T lymphocytes count (≤500 vs >500 cells/mm3) and HIV viral load (≤40 vs > 40 copies/mL). Values of *p* lower than 0.05 were considered statistically significant and a confidence interval (CI) of 95% was used to calculate the Prevalence Ratio (PR).

The association between exposure (Lymphocytes T CD4 count, HIV viral load and other factors) and outcome (HPV clearance and incidence) was available by hazard ratios (HR) with 95% CI, taking follow-up time into account. HR were estimated from Cox regression models to account for the matching for variables as age, parity, sexual debut, contraceptive oral combined use (COC use). The women who presented an incidence of different virus types were considered individually for each infection by type-specific virus in the statistical analysis. A Kaplan-Meier survival plot graphically illustrates the association between HPV clearance and HPV multiples types infection, HPV incidence and nulliparity, COC use and ART use.

## Results

Three hundred and thirty-eight women were included at the beginning of this study, and five were excluded from the analysis: three women asked to withdraw consent, stating that they had no spare time for the subsequent follow-up visits, one woman whose first sample was not processed, and one woman who died before the first follow-up visit (S1 Fig). Therefore, this study included 333 women. Of these subjects, 258 WLHA returned for the 6-month follow-up appointment (77.5% of the initial population), and 280 WLHA returned for the 12-month follow-up appointment (84.1% of the initial population).

### HPV prevalence

In this study, 33.3% (111/333) of WLHA were infected with HR-HPV at baseline, 5.1% (17/333) with HPV 16, 3.9% (13/333) with HPV 18 and 29.4% (98/333) with another HR-HPV strain. Amongst the evaluated population, 4.8% (16/333) WLHA showed infection with multiple types (HPV 16 and/or 18 and another HR-HPV) at baseline.

The mean age of the WLHA assessed at the time of inclusion was 37.9 (±11.0) years old. The mean age of sexual debut was at 16.3 (±3.3) years and the first pregnancy was at 19.8 (±4.5) years. Thirty WLHA (9.3% of the population studied) were pregnant at enrolment and 28 (8.4%) had never been pregnant before. After evaluating the WLHA who had been pregnant, 54.5% had a CD4^+^ T cells count ≤ 500 cells/mm^3^ (*p* = 0.001) and 63.3% had HIV viral load > 40 copies/mL (*p* = 0.001), showing clinical and viral differences between the non-pregnant WLHA.

**Fig 1:**
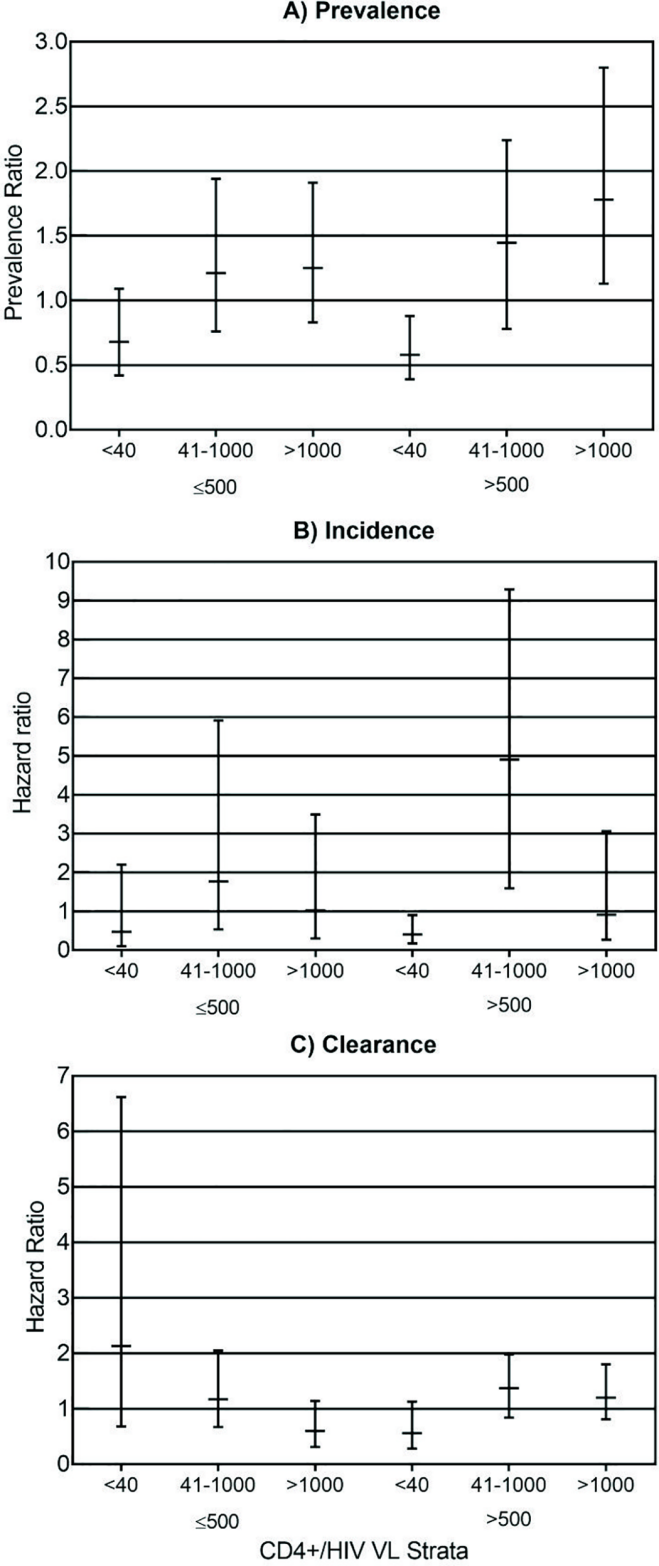
Prevalence, incidence and clearance of HR-HPV infection in women living with HIV/AIDS by CD4+ T-lymphocyte cells count and HIV VL strata. HIV VL was stratified in the following stratum: <40 copies/mL (undetectable), 41 to 1000 copies/mL and over 1000 copies/mL. CD4+ T-lymphocyte strata are as follow: ≤500 cells/μL and >500 cells/μL. The risk estimates are show in prevalence or hazard ratio and their confidence intervals. (A) Prevalent detection of HR-HPV is shown in prevalence ratio in a given HIV VL stratum relative to each CD4+ T cell count. (B) Incident HR-HPV infection risk was estimated using hazard ratio determined with Cox model for each HIV VL stratum in the different CD4+ T-cell count groups. (C) Clerance of HR-HPV infection risk was estimated using hazard ratio determined with Cox model for each HIV VL stratum in the different CD4+ T-cell count groups.

Among the WLHA evaluated, 82.9% (276/333) of women were using ART with a mean treatment duration of 81.3 (±65.3) months and 63.7% (211/331) had HIV VL ≤ 40 copies/mL. The mean CD4^+^ T cell count was 707.9 (±388.7) cells/mm^3^. The association between Lymphocytes T CD4, HIV viral load and HPV infection at baseline is described in Fig 1.

Regarding sexual activity at enrolment, 50 (15.0%) of WLHA reported not having an active sex life in the study period, with 30% (15/50) prevalence of HR-HPV in that group. Amongst the WLHA who had an active sex life, 77 (33.5%) had a partner living with HIV/AIDS, 114 (49.5%) had HIV non-infected partner, and 39 (17.0%) had a partner with unknown HIV status. Irregular condom use was reported by 111 (33.3%) of the WLHA, while 70 (21.0%) used depot medroxyprogesterone acetate (DMPA), 19 (5.7%) were using oral contraceptives, and 6 (1.8%) used monthly injectable contraceptives. CT infection was identified in 17 (5.1%) cervical samples and just one (0.3%) NG case was found.

Details on HPV infection at baseline and its association with sociodemographic, clinical, and behavioral characteristics are described in Table 1.

**Table 1:** Clinical and socio-demographic characteristics of 333 women living with HIV/AIDS followed in a cohort in northeastern Brazil, based on the presence of baseline HPV co-infection.

	Total	Baseline HPV [n (%)]	No HPV [n (%)]	*p*	PR* (95%CI)
***Socio-demographic***					
≤ 30 years	91	47 (42.0)	44 (19.9)	<0.01	1.92 (1.44–2.56)
Non-white ethnicity	311	108 (96.4)	203 (91.9)	0.16	1.91 (0.78–4.70)
Single	173	53 (47.3)	120 (54.3)	0.23	0.83 (0.61–1.12)
≤ 8 schooling years	121	37 (33.6)	84 (39.3)	0.32	0.85 (0.61–1.18)
Monthly income ≤ 2 MW^a^	273	100 (89.3)	173 (78.6)	0.02	1.80 (1.06–3.06)
Drug use	30	14 (12.5)	16 (7.2)	0.11	1.44 (0.95–2.19)
Alcohol use	175	63 (56.2)	112 (50.7)	0.34	1.16 (0.86–1.58)
Smoking	33	17 (15.5)	16 (7.3)	0.02	1.65 (1.14–2.39)
***Sex-risk behaviors***					
Sexual debut ≤ 16 years	187	71 (65.1)	116 (52.5)	0.03	1.43 (1.03–1.98)
Irregular condom use	111	39 (35.1)	72 (32.6)	0.64	1.08 (0.79–1.48)
> 3 lifetime sexual partners	218	75 (67.6)	143 (64.7)	0.60	1.09 (0.79–1.51)
Transactional sex	30	14 (12.5)	16 (7.2)	0.11	1.44 (0.95–2.19)
Alcohol before sex	138	46 (41.8)	92 (42.0)	0.97	1.00 (0.73–1.36)
Drugs before sex	24	13 (11.6)	11 (5.0)	0.03	1.69 (1.13–2.53)
***Clinical findings***					
CT co-infection	12	8 (7.1)	4 (1.8)	0.03	2.06 (1.34–3.16)
Pregnant	30	17 (15.6)	13 (6.1)	<0.01	1.80 (1.26–2.57)
COC pill use	19	9 (8.0)	10 (4.5)	0.19	1.44 (0.88–2.38)
DMPA use	70	25 (22.3)	45 (20.4)	0.68	1.08 (0.76–1.55)
Parity (>3)	47	14 (13.6)	33 (16.2)	0.55	0.87 (0.54–1.39)
Previous STI	160	56 (50.0)	104 (47.1)	0.61	1.08 (0.80–1.46)
Previous condyloma	81	30 (26.8)	51 (23.2)	0.47	1.13 (0.81–1.59)
No ART use	57	29 (25.9)	28 (12.7)	<0.01	1.69 (1.24–2.31)
≤ 1 year on ART	57	31 (36.5)	26 (13.5)	<0.01	2.22 (1.59–3.09)
≤ 1 year from HIV diagnosis	37	23 (20.5)	14 (6.3)	<0.01	2.07 (1.52–2.81)
CD4+ ≤ 500 cells/μL at baseline	97	46 (41.1)	51 (23.1)	<0.01	1.70 (1.27–2.27)
Viral load > 40 cp/mL at baseline	120	56 (50.5)	64 (29.1)	<0.01	1.79 (1.33–2.41)
Altered Pap test at baseline	35	21 (18.8)	14 (6.4)	<0.01	1.96 (1.42–2.70)
ASCUS at 1^st^ Pap test	14	6 (5.4)	8 (3.6)	0,46	1.29 (0.69–2.40)
LSIL at 1^st^ Pap test	16	10 (8.9)	6 (2.7)	0.01	1.94 (1.28–2.92)
HSIL at 1^st^ Pap test	5	5 (4.5)	0 (0)	<0.01	3.06 (2.62–3.57)
Altered colposcopy	38	22 (19.8)	16 (7.4)	<0.01	1.89 (1.37–2.60)

CT: *Chlamydia trachomatis*; COC: combined oral contraceptive; DMPA: Depot medroxyprogesterone acetate; ASCUS: atypical squamous cells of undetermined significance; LSIL: low-grade intraepithelial lesion; HSIL: high-grade intraepithelial lesion; STI: sexually transmitted infections; ART: antiretroviral therapy

^#^HPV infection by HPV 16, 18, 31, 33, 35, 39, 45, 51, 52, 56, 58, 59, 66 or 68

^a^MW: minimum wage ≈ $194

*PR: Prevalence Ratio

At the time of study inclusion, 36 altered oncotic cytologies were found, of which 14 (4.2%) were described as atypical squamous cells of undetermined significance (ASCUS), 17 (5.1%) were low-grade intraepithelial lesions (LSIL) and 5 (1.5%) were high-grade intraepithelial lesions (HSIL) (Table 2). At the 6-month follow-up visit, cervical smears showed 3 (1.2%) WLHA with HSIL, and no high-grade lesions were found at the 12-month follow-up visit. All WLHA who had an HSIL diagnosis by oncotic cytology had HR-HPV present in the HPV-DNA screening.

**Table 2:** Association between oncotic cytology, HPV genotype, virologic suppression of HIV, and immunologic response at baseline in a cohort of women living with HIV/AIDS, northeastern Brazil.

Oncotic cytology (N = 332)	HPV 16 [n (%)]	*p*	HPV 18 [n (%)]	*p*	Other HR-HPV^b^ [n (%)]	*p*	HPV Negative [n (%)]	*p*	CD4+ ≤350 cells/mm^3^ [n (%)]	*p*	HIV VL > 40 cp/mL [n (%)]	*p*
Negative (N = 296)	13 (4.4)	0.01	9 (3.0)	<0,01	78 (26.3)	<0.01	200 (69.7)	<0.01	31 (10.4)	0.24	99 (33.6)	<0.01
ASCUS (N = 14)	1 (7.1)		1 (7.1)		6 (42.9)		8 (57.1)		2 (14.3)		4 (28.6)	
LSIL (N = 17)	1 (6.3)		0 (0.0)		10 (62.5)		6 (37.5)		4 (26.7)		13 (81.3)	
HSIL (N = 5)	2 (40.0)		3 (60.0)		4 (80.0)		0 (0.0)		1 (20.0)		3 (60.0)	

ASCUS: atypical squamous cells of undetermined significance; LSIL: low-grade intraepithelial lesion; HSIL: high-grade intraepithelial lesion; HIV VL: HIV viral load

^b^ Other HR-HPV: HPV 31, 33, 35, 39, 45, 51, 52, 56, 58, 59, 66, and 68

### HPV incidence

**Fig 2:**
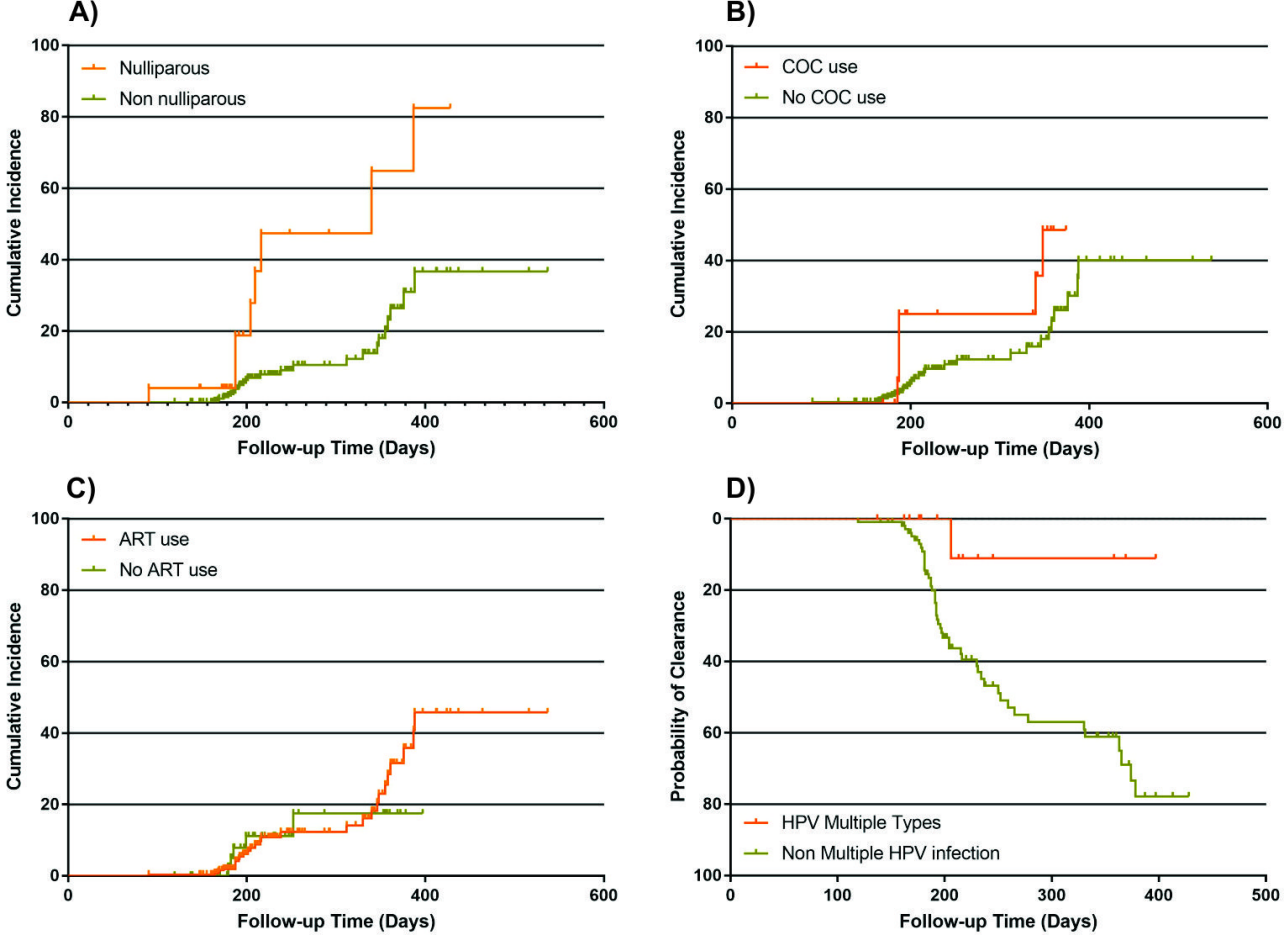
Kaplan-Meier estimates of the incidence and time to clearance of cervical HR-HPV infection in women living with HIV/AIDS by clinical and virological characteristics. (A) Incidence of HR-HPV by parity. (B) Incidence of HR-HPV by COC pill use. (C) Incidence of HR-HPV by ART use. (D) Clearance of HR-HPV by the presence of multiple HPV infection.

The incidence of HR-HPV infection during the follow-up was 10.8% (35/323). The 6-month incidence was 7.7% (25/323) and the 12-month incidence was 3.7% (12/323). The incidence of HPV 16 was 0.8% (2/247) in 6-month follow-up and 0.4% (1/270) in 12-month follow-up. At these appointment, the incidence of HPV 18 was 1.2% (3/252) and 1.9% (5/169) respectively. The incidence of other types HR-HPV was 11.5% (22/191) in 6-month follow-up and 3.2% (6/190) in the 12-month visit. The incidence at least one HPV infection during the follow-up was associated with nulliparity (*p* = 0.001; aHR 4.32; 95%CI 1.65–11.33), combined oral contraceptive use (*p* = 0.024; HR 2.82; 95%CI 1.15–6.94), and detectable HIV viral load at 6-month follow-up appointment (*p* = 0.054; aHR 2.35; 95%CI 1.08–5.13) (Fig 2). The association with parity and HIV viral load remained after the evaluation by Cox regression model. The association between CD4 T lymphocytes count, HIV viral load and HPV incidence is described in Fig 1.

The analysis of HR-HPV incidence and the women socio-demographic and clinical characteristic is described in Table 3.

**Table 3:** Risk factors for incident cervical HR-HPV infection in a cohort of women living with HIV/AIDS, Northeastern Brazil, 2013–2015.

	HR-HPV total incidence			
	[n(%)]		*p*	HR
	Yes	No		
***Incident HR-HPV*** ^a^				
HPV 16	-	-	0.27	Reference
HPV 18	-	-	0.45	1.60 (0.48–5.42)
Other HR-HPV^b^	-	-	0.19	0.54 (0.22–1.34)
***Socio-demographics***				
≤ 30 years	14 (41.2)	74 (25.7)	0.13	1.69 (0.85–3.56)
White ethnicity	3 (8.8)	19 (6.6)	0.99	1.01 (0.31–3.32)
Single	16 (47.1)	151 (52.4)	0.63	0.85 (0.43–1.66)
≤ 8 schooling years	13 (39.4)	102 (36.4)	0.62	1.19 (0.59–2.40)
Monthly income ≤ 2 MW^c^	15 (44.1)	92 (32.1)	0.73	1.17 (0.48–2.84)
Drug use	6 (17.6)	21 (7.3)	0.13	1.97 (0.81–4.77)
Alcohol use	22 (64.7)	148 (51.4)	0.22	1.56 (0.77–3.15)
Smoking	6 (17.6)	21 (7.3)	0.72	1.21 (0.42–3.47)
***Sex-risk behaviors***				
Sexual debut ≤ 16 years	26 (76.5)	153 (53.1)	0.07	2.11 (0.96–4.68)
> 3 lifetime sexual partners	23 (67.6)	188 (65.5)	0.92	1.04 (0.51–2.13)
Irregular condom use	15 (44.1)	92 (32.1)	0.21	1.55 (0.78–3.05)
Transactional sex	3 (8.8)	24 (8.3)	0.76	1.21 (0.37–3.97)
Alcohol before sex	14 (41.2)	120 (42.3)	0.56	0.82 (0.41–1.62)
Drugs before sex	1 (2.9)	20 (6.9)	0.26	0.32 (0.04–2.34)
***Clinical findings***				
Pregnant at inclusion	1 (2.9)	29 (10.1)	0.10	0.19 (0.03–1.37)
Parity (>3)	4 (15.4)	39 (14.4)	0.80	1.15 (0.39–3.37)
Nulliparous	8 (23.5)	20 (6.9)	<0.01	3.66 (1.65–8.11)
CT co-infection	1 (2.9)	9 (3.1)	1.00	0.98 (0.13–7.24)
COC pill use	6 (17.6)	12 (4.2)	0.02	2.82 (1.15–6.94)
DMPA use	5 (14.7)	64 (22.2)	0.72	0.84 (0.32–2.17)
Previous STI	16 (47.1)	139 (48.3)	0.80	0.92 (0.47–1.80)
Previous condyloma	13 (38.2)	69 (24.0)	0.31	1.43 (0.71–2.87)
No ART use	5 (14.7)	44 (15.3)	0.70	0.83 (0.32–2.15)
≤ 1 year on ART	31 (91.2)	256 (88.9)	0.93	1.04 (0.45–2.43)
≤ 1 year from HIV diagnosis	3 (8.8)	32 (11.1)	0.85	0.89 (0.27–2.93)
***Follow-up***				
CD4+ >500 cells/μL				
At baseline	11 (32.4)	79 (27.4)	0.73	1.18 (0.45–3.08)
6-month visit	7 (25.0)	57 (20.9)	0.09	0.47 (0.20–1.12)
12-month visit	11 (37.9)	65 (24.3)	0.45	0.69 (0.26–1.82)
HIV viral load >40 cp/mL				
At baseline	20 (58.8)	94 (32.6)	0.89	0.55 (0.28–1.09)
6-month visit	16 (55.2)	80 (30.0)	0.05	2.06 (0.99–4.29)
12-month visit	10 (35.7)	74 (26.8)	0.81	0.91 (0.42–1.98)
Altered oncotic cytology				
At baseline	2 (5.9)	33 (11.5)	0.31	0.47 (0.11–1.98)
6-month visit	3 (11.1)	20 (8.9)	0.83	2.06 (0.99–4.29)
12-month visit	1 (3.8)	18 (8.0)	0.36	0.39 (0.05–2.90)
Altered colposcopy				
At baseline	4 (11.8)	33 (11.6)	0.42	1.55 (0.54–4.41)
6-month visit	4 (13.8)	32 (13.0)	0.70	1.24 (0.42–3.62)
12-month visit	3 (9.7)	25 (9.3)	0.68	1.28 (0.39–4.25)

CT: *Chlamydia trachomatis*; COC: combined oral contraceptive

^a^ Incident HR-HPV: New high-risk HPV infection

^b^ Other HR-HPV: HPV 31, 33, 35, 39, 45, 51, 52, 56, 58, 59, 66 and 68

^c^MW: minimum wage ≈ $194

### HPV clearance

Clearance of HR-HPV infection occurred in 41.7% (50 of 120) of the infections. Women older than 30 years at baseline (35 of 70) had a higher number of HPV infection elimination (*p* = 0.028; OR 2.33; 95%CI 1.09–5.02), this association did not remain as significant after the evaluation by Cox regression model. Women with CD4+ T lymphocyte count >500 cells/mm^3^ at baseline, at 6 and 12-month follow-up appointment (aHR 2.27; 95%CI 1.17–4.43; HR 3.51 95%CI 1.62–7.64 and 2.64 95%CI 1.18–5.90) were significantly more likely to clear the HR-HPV infection. The association between Lymphocytes T CD4, HIV viral load and HPV clearance is described in Fig 1.

At the Cox regression model, the clearance occurred less frequently among women who had altered colposcopy (at baseline, HR 0.38 95%CI 0.16–0.90; and 1-year follow-up: HR 0.25, 95%CI 0.08–0.79) during the subsequent visits. The women infected with multiple HR-HPV at baseline were less likely to clear the infection (HR 0.14 95% CI 0.02–1.02). There was no association between HR-HPV clearance and abnormal findings at the oncotic cytology (Table 4).

**Table 4:** Risk factors related to clearance of cervical HR-HPV infection in a cohort of women living with HIV/AIDS, Northeastern Brazil, 2013–2015.

	HR-HPV clearance			
	[n (%)]		*p*	HR
	Yes	No		
***HR-HPV*** ^a^				
HPV 16	5 (10.2)	11 (15.7)	0.94	0.97 (0.38–2.45)
HPV 18	3 (6.1)	10 (14.3)	0.13	0.41 (0.13–1.30)
Other HR-HPV^b^	36 (73.5)	58 (82.9)	0.10	0.58 (0.31–1.10)
Multiple HR-HPV^c^	1 (2.0)	14 (20.0)	0.05	0.14 (0.02–1.02)
***Socio-demographics***				
> 30 years	35 (70.0)	35 (50.0)	0.29	1.39 (0.75–2.59)
Single	23 (46.0)	33 (47.1)	0.63	0.87 (0.50–1.52)
Monthly income ≤ 2 MW^d^	19 (38.8)	26 (37.1)	0.64	0.84 (0.39–1.78)
Drug use	4 (8.0)	10 (14.3)	0.63	0.78 (0.28–1.16)
Alcohol use	31 (62.0)	39 (55.7)	0.14	1.55 (0.87–2.78)
Smoking	6 (12.2)	12 (17.4)	0.27	0.62 (0.26–1.46)
***Sex-risk behaviors***				
Sexual debut ≤ 16 years	32 (64.0)	45 (64.3)	0.98	0.99 (0.56–1.78)
> 3 lifetime sexual partners	33 (66.0)	46 (66.7)	0.52	0.82 (0.46–1.48)
Irregular condom use	19 (38.8)	29 (37.1)	0.53	1.20 (0.68–2.14)
Transactional sex	4 (8.0)	10 (14.3)	0.38	0.63 (0.23–1.76)
***Clinical findings***				
Pregnant at inclusion	4 (8.0)	13 (18.6)	0.15	0.47 (0.17–1.32)
Parity (>3)	6 (14.0)	6 (9.2)	0.24	1.68 (0.71–4.00)
Nulliparous	8 (16.0)	5 (7.1)	0.77	1.98 (0.93–4.24)
CT co-infection	3 (6.0)	4 (5.7)	0.88	1.10 (0.34–3.54)
COC pill use	5 (10.0)	5 (7.1)	0.62	1.27 (0.50–3.21)
Previous STI	24 (48.0)	36 (51.4)	0.79	0.93 (0.53–1.62)
Previous condyloma	13 (26.0)	21 (30.0)	0.79	0.92 (0.49–1.72)
No ART use	12 (29.3)	20 (37.7)	0.17	0.60 (0.29–1.25)
> 1 year on ART	29 (77.8)	33 (62.3)	0.43	1.31 (0.69–2.57)
> 1 year from HIV diagnosis	44 (88.0)	52 (74.3)	0.12	1.99 (0.85–4.69)
***Follow-up***				
CD4+ >500 cells/μL				
At baseline	37 (74.0)	35 (50.0)	<0.01	2.92 (1.54–5.54)
6-month visit	36 (81.8)	33 (52.4)	<0.01	3.51 (1.62–7.64)
12-month visit	41 (85.4)	41 (63.1)	0.02	2.64 (1.18–5.90)
HIV viral load >40 cp/mL				
At baseline	23 (46.0)	37 (52.9)	0.32	0.75 (0.43–1.32)
6-month visit	16 (36.4)	31 (49.2)	0.11	0.61 (0.33–1.13)
12-month visit	15 (31.2)	27 (41.5)	0.12	0.61 (0.88–1.13)
Altered oncotic cytology				
At baseline	7 (14.0)	14 (20.0)	0.36	0.69 (0.31–1.53)
6-month visit	7 (16.3)	7 (14.9)	0.53	1.30 (0.57–2.95)
12-month visit	4 (9.1)	8 (15.7)	0.21	0.52 (0.18–1.45)
Altered colposcopy				
At baseline	7 (14.0)	18 (25.7)	0.03	0.38 (0.16–0.90)
6-month visit	7 (15.9)	17 (31.5)	0.08	0.49 (0.22–1.10)
12-month visit	3 (6.2)	17 (27.0)	0.02	0.25 (0.08–0.79)

CT: *Chlamydia trachomatis*; COC: combined oral contraceptive

^a^ HR-HPV: high-risk HPV

^b^ Other HR-HPV: HPV 31, 33, 35, 39, 45, 51, 52, 56, 58, 59, 66 and 68

^c^ Multiple HR-HPV: infection with HPV 16 and/or HPV 18 and other HR-HPV

^d^MW: minimum wage ≈ $194

## Discussion

The prevalence of HR-HPV among WLHA at baseline was 33.3%, with an HPV 16 prevalence of 5.1% and HPV 18 prevalence of 3.9%. These rates corroborate the findings of other studies carried out at Brazil, which have shown an HR-HPV prevalence of 32%-65% among women living with HIV/AIDS [20–22]. According to some authors, prevalence rates of HPV are higher among WLHA than in the general population [23,24].

Persistent infection with HR-HPV is necessary for the progression of cervical intraepithelial lesions to cervical cancer [7]; therefore, research into HR-HPV persistence and clearance has been proposed as a key strategy to define the populations that need better monitoring and follow-up [25]. We found a 41.7% rate of clearance of HR-HPV infection during follow-up. Some study related that the mean time for HPV clearance is 6–12 months in women HIV non-infected [26,27], in our study, among the women who had cleared the infection, 82.0% clearance occurred in 6-months follow-up. A lower CD4+ T lymphocyte count during the follow-up of WLHA was associated with a higher prevalence and with decreased clearance of HR-HPV. These findings are consistent with those provided by a study carried out in Brazil, which showed a greater chance of progression to HSIL among WLHA who presented compromised immunity, positive screening for HPV DNA and an abnormal cervical smear[28]. Immunological changes associated with HIV infection contribute to the pathogenesis of HPV through a marked reduction in the cellular immune response, which prevents the resolution of HPV-related lesions, as well as the contribution of HIV to the persistence of the infection and increased viral replication of HPV [29–31]. However, these data are still controversial. In a study that evaluated the impact of ART on HPV infection among adolescents living with HIV, the CD4+ T count had no significant impact on the prevalence or incidence of HPV [32].

The present study shows an incidence of at least one type of HR-HPV of 10.6% at the sixth month and 6.5% in one year. HPV 18 had an incidence of 1.8% during the cohort, higher than the incidence of HPV 16 (1.1%). A recent study carried out in Brazil identified an incidence of 18.5% for new cases of HPV (about 34 viral types) among WLHA, of which 9.2% were HPV 16 [28]. Among women without HIV infection in the Netherlands, the HPV 16 incidence was 6.8% [33]. Non-16/18 HR-HPV genotypes are more frequent among WLHA than in the general population [19]. Multicentric studies on the prevalence of HPV among WLHA in developing countries are needed to evaluate the effectiveness of the vaccination coverage available for this population.

When assessing the age of the study participants, we identified a higher prevalence and incidence of at least one type of HR-HPV among the younger WLHA than among those over 30 years of age. This association has been largely described among WLHA and the general population [23,34]. However, the evaluation of the clearance of HR-HPV infection showed the most cases of clearance in older women. This finding is conflicting to the described in a previous studies on WLHA, which showed younger women as being more likely to clear the infection [35,36]. HPV 16 was more frequently cleared among women over 30 years old (57.1% versus 11.1%) than by younger WLHA. According to previous studies, unlike other HR-HPV types, infection with HPV 16 is not susceptible to the interference from immune reconstitution with ART use, and remains persistent [35,37]. In the population presented here, HPV 16 clearance occurred among women over 30 years with CD4+ T count >500cells/mm^3^. The presence of HPV 16 variants according to the ethnicity and geographic origin of the population has been a subject of study throughout the world [38]. This fact could explain the difference in the persistence of the infection in our population; however, this evaluation was not carried out in this study.

In our study, the prevalence and incidence of at least one genotype of HR-HPV were significantly associated with some already well-established co-factors related to oncogenesis, such as the use of combined oral contraceptives and smoking [39]. A recent cohort study with more than 300,000 women has indicated that prolonged exposure to endogenous and exogenous hormones, such as combined oral contraceptives, is related to cervical carcinogenesis [40]. Smoking interferes with cell cycle regulation and with the release of free radicals and it is involved in the oxidative stress process that occurs with chronic inflammation. Chronic inflammation is also promoted by hormonal action in the context of prolonged use of oral contraceptives and increased parity, which allows exposure of the transformation zone of the ectocervix for a longer period of time; these factors are also associated with a higher prevalence of HPV infection [8,41,42].

Endocervical infection with CT is considered a cofactor for precursor lesions for cervical cancer. In the studied population, we have found an association between CT infection with a higher prevalence of at least one type of HR-HPV, as shown in a study with over 800 women who were screened for cervical cancer [43]. Changes promoted by the inflammatory process, such as cytokine release and locally produced free radicals in cervicitis are related to the persistence of HR-HPV infection [44,45]. The data presented here in do not show an association between CT infection and the incidence of HR-HPV, perhaps because treatment for CT infection was performed shortly after its diagnosis.

The increased prevalence of HR-HPV was associated with the absence of ART use, a shorter period of antiretroviral treatment and shorter time from HIV diagnosis; however, this association did not maintain significance in the evaluation of HR-HPV clearance and incidence. A detectable VL (>40 copies/mL), however, was associated with increased prevalence and incidence of infection with at least one type of HR-HPV. The diversity of study designs applied is one of the key issues that hinder the construction of consistent data about the impact of ART use and viral suppression of HIV on HPV infection. Therefore, data on the association of ART with abnormalities in oncotic cytology and its influence on the progression of precursor lesions are still controversial [4,10]. Recent studies have demonstrated that HIV viral suppression seems to change the course of HPV infection in WLHA [11,12,46]. An early start to ART, as recommended by the World Health Organization (WHO) in 2012 and by the HMB since 2013[47,48], may change this scenario, since starting treatment with a high CD4+ T lymphocyte count and no other significant alterations to the immune system may contribute to HR-HPV clearance, as well as a reduction in HSIL and cervix cancer incidence, therefore providing better outcomes for WLHA.

HSIL diagnosis in WLHA at baseline was associated with the presence of high risk HPV-DNA in all cases in this study. Some authors have suggested using molecular HPV diagnostics as a marker for populations that need more careful screening [49]. The faster progression to cervical cancer in WLHA shows the need for tests with higher sensitivity for the effective prevention of this disease [9]. Identifying the clinical and sociodemographic characteristics of WLHA with higher HPV prevalence contributes to secondary prevention and increased surveillance, thus making it possible to diagnose and treat high-grade lesions early. A morphologic diagnosis using cytology and histology has a fundamental role in therapy and follow-up, and remains an important step in screening protocols all over the world [17,50,51].

There were limitations to this study and, initially, we encountered difficulty during follow-up, despite phone scheduling, which was used to make contact and to confirm appointments. This limitation had a smaller impact due to the sample size calculation, which predicted a follow-up drop-out of 18%; we had 84.1% of the WLHA return for their 12-month appointments. Another limitation refers to the fact that the assay for HPV-DNA diagnostics differentiates HPV 16 from HPV 18, responsible for 70% of cervix cancer cases, but does not differentiate the remaining 12 constant HR-HPV in the pool. The short follow-up period of this study is also a limitation in terms of assessing HSIL evolution, following the clearance of incident cases or reactivation of a latent HPV infection. The HIV viral load was not available at cervical fluid, to clarify the interactions between HPV and HIV in genital tract epithelium, more research is necessary.

Our results suggest that using HPV-DNA as a screening tool for HPV infection among WLHA, allied to morphological exams, would allow for organized access to methods for the early diagnosis and prevention of cervical cancer in this population. The continuous WLHA follow-up in our service will contribute with more information about the interaction between HPV and HIV infection. Identifying modifiable risk factors of HPV infection, such as combined oral contraceptive use, smoking, and HIV viral suppression allow for changes in the current scenario. This study made it possible to define the group of women who need a more careful screening and described the association between the immune response and HIV viral suppression with a reduction in the incidence of and an increase in the clearance of HR-HPV.

## Supporting information

S1 Fig Flowchart of the cohort of women living with HIV/AIDS in Salvador, Brazil. This is the flowchart of women living with HIV/AIDS assisted during the cohort study.
(DOCX)

S1 Appendix Questionnaire of the cohort of women living with HIV/AIDS in Salvador, Brazil. This is the questionnaire model applied during the interviews conducted in the study.
(DOCX)
