# Supplementary material for: Predictors of HPV incidence and clearance in a cohort of Brazilian HIV-infected women
Source: PLoS One. 2017 Oct 5;12(10):e0185423. doi: 10.1371/journal.pone.0185423 (PMC5628817; doi:10.1371/journal.pone.0185423)
Supplement: S1 Fig — This is the flowchart of women living with HIV/AIDS assisted during the cohort study. (DOCX) [file pone.0185423.s001.docx]

S1 Figure. Flowchart of the cohort of WLHA in Salvador, Brazil
