# Supplementary material for: Predictors of HPV incidence and clearance in a cohort of Brazilian HIV-infected women
Source: PLoS One. 2017 Oct 5;12(10):e0185423. doi: 10.1371/journal.pone.0185423 (PMC5628817; doi:10.1371/journal.pone.0185423)
Supplement: S1 Appendix — This is the questionnaire model applied during the interviews conducted in the study. (DOCX) [file pone.0185423.s002.docx]

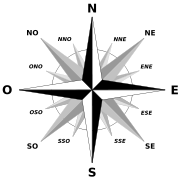
**ROSA GROUP**

**Women Questionnaire**

**Genital Infection and HPV in a woman living with HIV cohort: the role of antiretroviral therapy and immune system response.**

**Questionnaire No.__________ Visit _______ Record N°_____________**

**Date of Interview ____/____/_____ Interviewer___________________**

1. Skin colour:

🞏Yellow (oriental) 🞏White (Caucasian) 🞏Indian 🞏Brown (mixed-race)🞏Black

1. Marital Status:

🞏Single 🞏Living with the partner 🞏Married 🞏Widowed 🞏Divorced/separated

1. Schooling:

🞏unschooled

🞏pre-school school incomplete

🞏primary school incomplete

(<4years)

🞏primary school complete / secondary school incomplete (<8years)

🞏middle school complete / higher education incomplete (<11years)

🞏higher education complete (>11years)

1. Profession/occupation:______________________
2. Mean monthly family income (MW-minimum wage 2013: R$678,00):_________

🞏< 1 MW 🞏 1-2 MW 🞏3-4 MW 🞏> = 5 MW

1. Religion:

🞏 No religion

🞏 Catholic

🞏 Protestant

🞏 Evangelical

🞏 Spiritism

🞏African-brasilian

🞏 Jehovah's Witness

🞏 Not defined

🞏Others____________

1. Previous Antiretroviral Therapy: 🞏PEP 🞏VT Prophylaxis 🞏Yes 🞏No
2. Time of use: ____days____months____years
3. Scheme:____________________________________________________
4. Pharmacy of withdraw: ________________________________________
5. HIV Diagnosis:____/____/____
6. Was the woman breastfed? 🞏 Yes 🞏 No 🞏 Does not know
7. 1ª menstrual period: _____ years old
8. Contraceptive methods **before** HIV diagnosis:

🞏 Contraceptive pill

🞏 Monthly contraceptive injections

🞏 Quarterly contraceptive trimestral

🞏 Contraceptive implant

🞏IUD

🞏Tubal ligation

🞏Condom

🞏Abstinence

🞏Vasectomy (Partner)

🞏None

🞏Non applicable (VT)

11. **Current** contraceptive method:

🞏 Contraceptive pill

🞏 Monthly contraceptive injections

🞏 Quarterly contraceptive trimestral

🞏 Contraceptive implant

🞏IUD

🞏Tubal ligation

🞏Condom

🞏Abstinence

🞏Vasectomy (Partner)

🞏None

12. Age at first pregnancy: _____ years old 🞏Never got pregnant

13. Parity: Gravida:_____ Delivery:______ Abortion:______

14. Deliveries: _____Vaginal ____Cesarean ____Forceps

**15. IF THE WOMAN IS PREGNANT:**

- 1. Gestational age at the exam collection:_____weeks

16. Alcohol use:

🞏 ______ times/week 🞏weekends only 🞏never 🞏rare occasions

17. Tobacco use:

🞏Yes 🞏_____Cigars/day

🞏No

🞏abstemious for: _____months____years

18. Drug use:

1. **Before** HIV diagnosis:

🞏does not use

🞏marijuana

🞏shooting cocaine

🞏cocaine (powder)

🞏crack

🞏amphetamines

🞏ecstasy

🞏solvent (inhaling)

🞏abstemious for __months__years

🞏non applicable (VT)

1. **After** HIV diagnosis:

🞏does not use

🞏marijuana

🞏shooting cocaine

🞏cocaine (powder)

🞏crack

🞏amphetamines

🞏ecstasy

🞏solvent (inhaling)

🞏abstemious for __months__years

19. Condom use:

🞏  **Before** HIV diagnosis:

🞏never 🞏sometimes 🞏always 🞏abstemious 🞏non applicable (VT)

🞏  **After** HIV diagnosis:

🞏never 🞏sometimes 🞏always 🞏abstemious 🞏non applicable (VT)

20. 1^st^ sexual relation: at _____years old 🞏sexual violence

21. Number of partners throughout life:_____

22. Current number of partners:_____

23. Regarding current partners, how many HIV+:_____ 🞏unknown

24. Current partners:

🞏 (1) regular, for: ____days____months _____years

🞏 (2) regular, for: ____days____months _____years

🞏 (1) eventual, for: ____days____months _____years

🞏 (2) eventual, for: ____days____months _____years

25. Have had sexual intercourse with:

🞏 Men 🞏 Women 🞏Both

26. Sexual practice:

🞏Vaginal 🞏Receptive/passive 🞏Insertive/active 🞏Never had sex

🞏Anal 🞏 Receptive/passive 🞏Insertive/active 🞏Never had sex

🞏Oral 🞏 Receptive/passive 🞏Insertive/active 🞏Never had sex

27. Alcohol use before sexual relation: 🞏Yes 🞏No

Use: 🞏eventually 🞏frequently

28. Drug use before sexual relation: 🞏Yes 🞏No

Use: 🞏eventually 🞏frequently

29. Sex for money: 🞏Yes 🞏No

30. Sex for drugs: 🞏Yes 🞏No

31. Last sexual relation: ___days ago, or ___weeks ago, or___months ago, or___years ago.

32. Ever had any STDs? 🞏Yes 🞏No

33. Which of the following?

🞏HPV/condyloma 🞏Herpes 🞏Syphilis 🞏Hepatitis B 🞏Other____________

34. Signs and symptoms (currently):

Vaginal/urethral discharge: 🞏Yes 🞏No

Ulcer: 🞏Yes 🞏No

Pelvic pain: 🞏Yes 🞏No

Fever: 🞏Yes 🞏No

Vaginal itching: 🞏Yes 🞏No

Dyspareunia: 🞏Yes 🞏No 🞏abstinence

Genital warts: 🞏Yes 🞏No

Anal pain: 🞏Yes 🞏No

**Physical Examination:**

Date:____/____/_____

Professional Responsible (Initials):______________________________

Vaginal/urethral discharge: 🞏Yes 🞏No

Genital ulcer: 🞏Yes 🞏No

Fever: 🞏Yes 🞏No

Cervicitis: 🞏Yes 🞏No

Bullous lesion: 🞏Yes 🞏No

Genital warts: 🞏Yes 🞏No

Abnormal TZ in cervix: 🞏Yes 🞏No

Pain at pelvic exam: 🞏Yes 🞏No 🞏Not performed
